# Trapping of 5-Fluorodeoxyuridine Monophosphate by Thymidylate Synthase Confers Resistance to 5-Fluorouracil

**DOI:** 10.1021/acsomega.1c06394

**Published:** 2022-02-09

**Authors:** Chinatsu Kurasaka, Nana Nishizawa, Yoko Ogino, Akira Sato

**Affiliations:** † Department of Biochemistry and Molecular Biology, Faculty of Pharmaceutical Sciences, Tokyo University of Science, 2641 Yamazaki, Noda, Chiba 278-8510, Japan; ‡ Department of Gene Regulation, Faculty of Pharmaceutical Sciences, Tokyo University of Science, 2641 Yamazaki, Noda, Chiba 278-8510, Japan

## Abstract

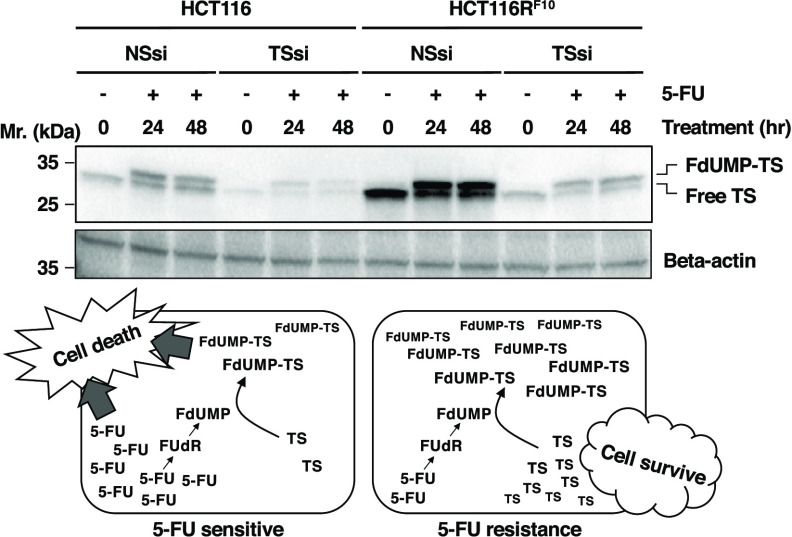

The major metabolite of the anticancer agent 5-fluorouracil (5-FU) is 5-fluorodeoxyuridine monophosphate (FdUMP), which is a potent inhibitor of thymidylate synthase (TS). Recently, we hypothesized that 5-FU-resistant colorectal cancer (CRC) cells have increased levels of TS protein relative to 5-FU-sensitive CRC cells and use a fraction of their TS to trap FdUMP, which results in resistance to 5-FU. In this study, we analyzed the difference between the regulation of the balance of the free, active form of TS and the inactive FdUMP-TS form in 5-FU-resistant HCT116 cells and parental HCT116 cells. Silencing of *TYMS*, the gene that encodes TS, resulted in greater enhancement of the anticancer effect of 5-FU in the 5-FU-resistant HCT116R^F10^ cells than in the parental HCT116 cells. In addition, the trapping of FdUMP by TS was more effective in the 5-FU-resistant HCT116R^F10^ cells than in the parental HCT116 cells. Our observations suggest that the regulation of the balance between the storage of the active TS form and the accumulation of FdUMP-TS is responsible for direct resistance to 5-FU. The findings provide a better understanding of 5-FU resistance mechanisms and may enable the development of anticancer strategies that reverse the sensitivity of 5-FU resistance in CRC cells.

## Introduction

5-Fluorouracil (5-FU) is a key anticancer drug used for the chemotherapy of colorectal cancer (CRC).^1,2^ In the body, 5-FU is converted to 5-fluorodeoxyuridine monophosphate (FdUMP), which is a potent inhibitor of thymidylate synthase (TS).^2−4^ TS, encoded by the *TYMS* gene in humans, catalyzes the conversion of dUMP to dTMP using the co-substrate 5,10-methylenetetrahydrofolate (CH2-THF).^5^ FdUMP forms a covalent ternary complex with TS and CH2-THF.^1,2,4,6−8^ This covalent ternary complex inhibits TS, depletes the intracellular dTTP pool, and subsequently inhibits DNA synthesis.^1−4^ In addition, 5-FU can exert cytotoxic effects through its incorporation into DNA and RNA as fluorodeoxyuridine triphosphate (FdUTP) and fluorouridine triphosphate (FUTP), respectively.^1−3^

Cancer cells are known to acquire resistance to anticancer drugs through a variety of mechanisms. The common cancer resistance mechanisms include inactivation of drugs, enhancement of drug efflux, alteration of drug target molecules, utilization of bypass pathways, facilitation of DNA damage repair, and escaping cell death.^1,2,9^ Many studies have examined the mechanisms of resistance to 5-FU and its derivatives.^1,2,9^ The function and/or expression of TS and other enzymes related to the 5-FU anabolism or catabolism pathways are often altered, accelerating resistance to 5-FU.^1,2,9−11^ In addition, the known mechanisms of 5-FU resistance are perturbance of cell death and autophagy, altered epigenetic repression, and expression/functional changes in drug transporters and noncoding RNA (i.e., microRNA and long noncoding RNA).^1,2,9^ It is widely considered that TS is part of an important molecular mechanism that enhances 5-FU sensitivity and that targeting TS is an excellent strategy to reverse 5-FU resistance.^1,2,12^ Indeed, numerous studies have shown that the gene amplification of *TYMS*, leading to mRNA and protein overexpression is a major mechanism of resistance to 5-FU and its derivatives.^12−15^ In addition, we have shown that 5-FU-resistant CRC cells increase *TYMS* expression relative to 5-FU-sensitive CRC cells and use a fraction of TS to trap FdUMP, which results in resistance to 5-FU and its derivatives.^16^ We predict that the regulation of TS status, which refers to the balance between the active free-TS form and the inactive FdUMP-TS covalent complex, may confer 5-FU resistance.^16^

In this study, we investigated the anticancer sensitivity of the 5-FU-resistant HCT116 cells and the parental HCT116 cells to 5-FU after *TYMS* knockdown. In addition, we analyzed the difference in the regulation of the balance between the active free-TS form and the inactive FdUMP-TS form in 5-FU-resistant HCT116 cells and the parental HCT116 cells. We discussed the possibility of the FdUMP trapping by the TS protein as one of the mechanisms of 5-FU resistance.

## Results

### Knockdown of *TYMS* Enhances the Anticancer Effect of 5-FU on 5-FU-Resistant HCT116R^F10^ Cells Compared with the Effect on Parental HCT116 Cells

**Figure 1:**
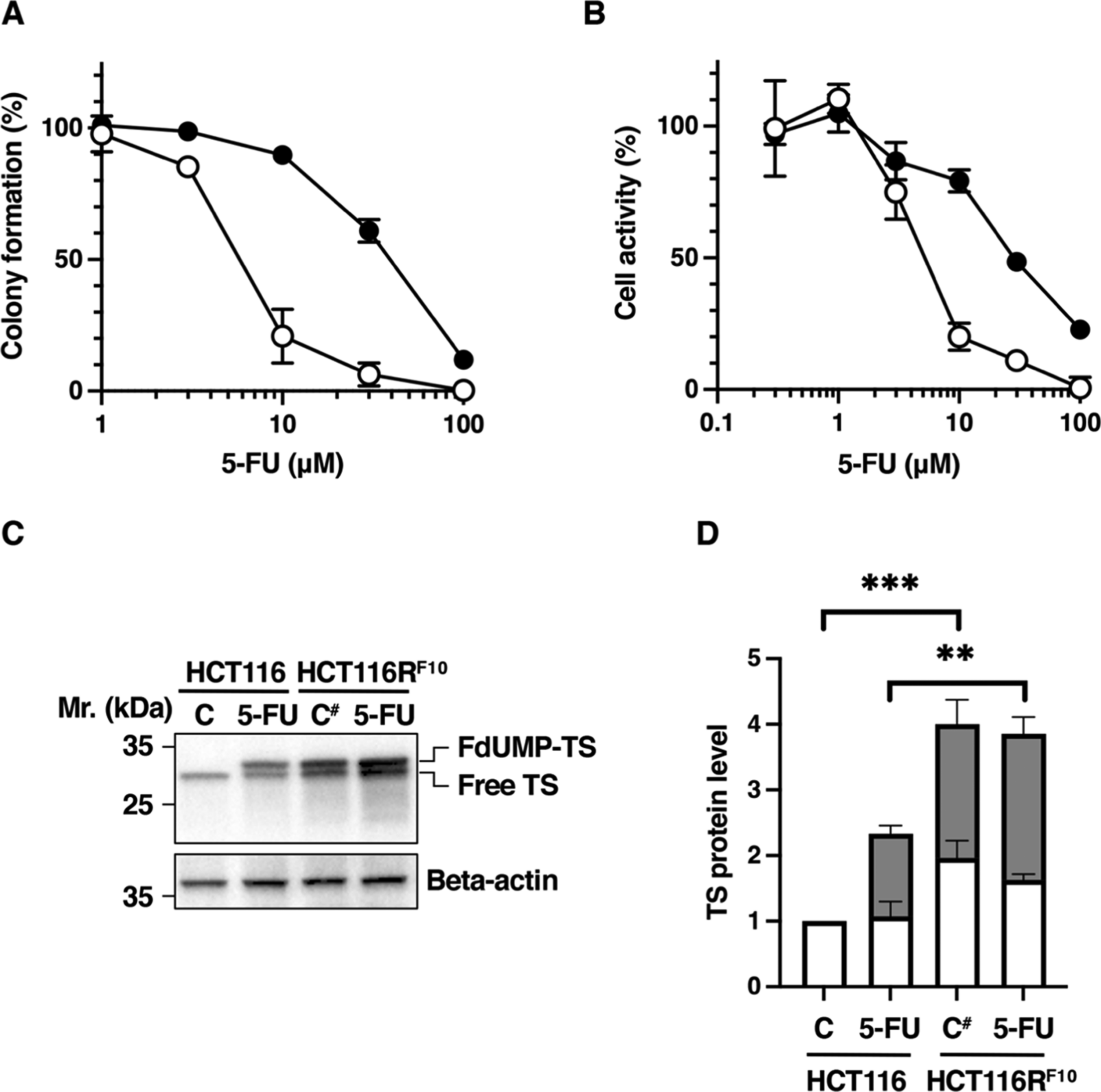
Two TS protein forms, free-TS and FdUMP-TS, are higher in 5-FU-resistant HCT116R^F10^ cells than in 5-FU-sensitive parental HCT116 cells. (A) 5-FU sensitivity of HCT116 and HCT116R^F10^ cells using colony formation assay. The cells were treated with the indicated concentration of 5-FU and incubated for 10 d. Colony formation (%) represents the average of three independent experiments, with error bars showing the ±SE (standard error) of triplicates. Solid circle, HCT116R^F10^ cells; open circle, HCT116 cells. (B) Cells were tested for cell activity after 72 h of treatment with the indicated concentration of 5-FU. Results represent the averages of three independent experiments, with error bars showing the ±SE of triplicates. (C) Protein levels of TS and β-actin in HCT116R^F10^ and HCT116 cells. Whole-cell lysates were prepared from parental HCT116 and HCT116R^F10^ cells. The expression levels of β-actin were used as an internal control. The data represent at least three independent experiments. (D) Protein levels of two TS forms, free-TS and FdUMP-TS, in HCT116 and HCT116R^F10^ cells. TS protein levels in HCT116R^F10^ cells are shown by the ratio of TS density to β-actin density relative to the control value for HCT116 cells. Results represent the average of three independent experiments, with error bars showing the ±SE of triplicates. C, control; passage culture condition of parental HCT116 cells (no drug or solvent). C#, passage culture condition of 5-FU-resistant HCT116R^F10^ cells; the cells were continually treated with 10 μM 5-FU. 5-FU, the cells were treated with 100 μM 5-FU for 24 h. White bar, free-TS form; gray bar, FdUMP-TS form. Student’s *t*-test, **p < 0.01 and ***p < 0.001. One-way analysis of variance (ANOVA), *p* < 0.0001 (for each total TS and FdUMP-TS levels of all groups).

The main anticancer mechanism of 5-FU is inhibiting TS by FdUMP, an active metabolite of 5-FU.^1,2,17^ The fundamental mechanism for this activity, proposed by Santi in 1980,^4^ is that FdUMP forms a covalent ternary complex with TS and CH2-THF.^4^ We have investigated the mechanisms of resistance to 5-FU in human CRC cell models, 5-FU-resistant HCT116R^F10^ cells, and parental HCT116 cells, revealing their genetic background by exome analysis. The concentration that confers 50% efficacy (EC_50_) of 5-FU in the 5-FU-resistant HCT116R^F10^ and parental HCT116 cells in the colony formation and WST-8 assays is shown in Table 1 and Figures 1A,B. We recently hypothesized that 5-FU-resistant CRC cells have upregulated TYMS expression and use a fraction of their TS to trap FdUMP, resulting in 5-FU resistance.^16^ Indeed, the protein levels of free-TS, FdUMP-TS-CH2-THF covalent complex, and total TS were significantly higher in HCT116R^F10^ cells than in HCT116 cells under the passage culture conditions (Figures 1C,D). Additionally, the protein levels of free-TS (native enzyme), FdUMP-TS covalent complex (which we termed as FdUMP-TS), and total TS were individually about 1.6–1.8-fold higher in HCT116R^F10^ cells than in HCT116 cells after treatment with 100 μM 5-FU for 24 h. In these experiments, we tested 5-FU at a concentration of 100 μM, which has a sufficient anticancer effect in HCT116R^F10^ and HCT116 cells. Interestingly, the total TS and FdUMP-TS levels were upregulated about twofold in HCT116 cells but not in HCT116R^F10^ cells after treatment with 5-FU for 24 h compared with individual subculture conditions. These results indicate that the 5-FU-resistant HCT116R^F10^ cells may have a system that traps FdUMP with TS and removes FdUMP-TS as a resistance mechanism.

**Table 1:** Summary of 5-FU Sensitivities in the 5-FU-Resistant HCT116R^F10^ Cells and Parental HCT116 Cells

	EC_50_ (μM)	
cell line	colony formation	WST-8
HCT116	5.5	5.1
HCT116R^F10^	38.0	29.0

**Figure 2:**
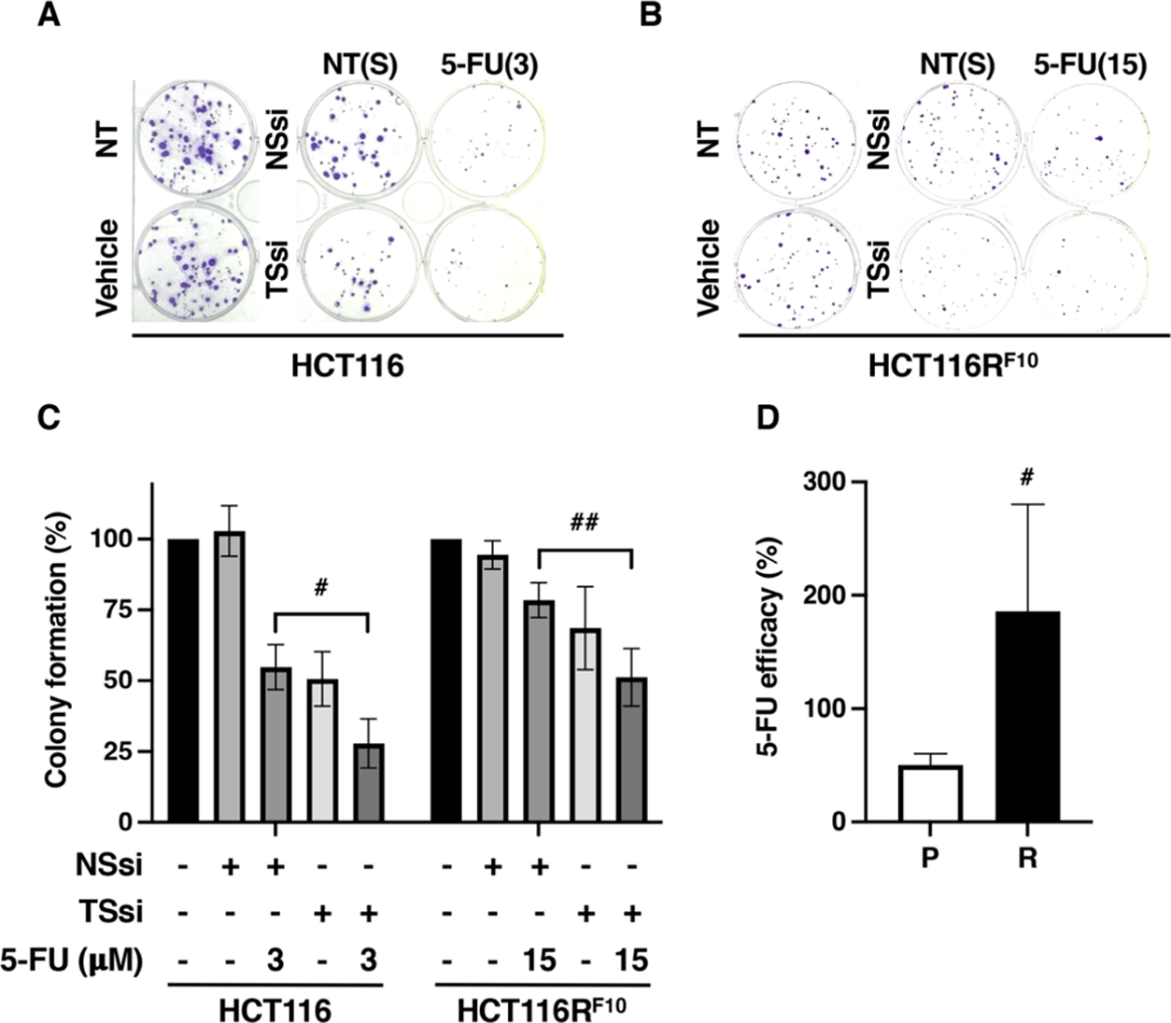
*TYMS* knockdown results in stronger enhancement of the anticancer activity of 5-FU in HCT116R^F10^ cells than in HCT116 cells. (A) Image of colony formation in HCT116 cells. (B) Image of colony formation in HCT116R^F10^ cells. Anticancer activity of 5-FU in HCT116R^F10^ cells and HCT116 cells, measured using the colony formation assay. HCT116R^F10^ cells and HCT116 cells were transfected with *TYMS*-targeted siRNA or nonsilencing siRNA. Then, both types of cells were treated with the indicated concentration of 5-FU and incubated for 9 days. NT, nontreatment; vehicle, lipofectamine RNAiMax alone; NT(S), solvent (dimethyl sulfoxide (DMSO)); NSsi, nonsilencing siRNA; TSsi, *TYMS*-targeted siRNA; 5-FU(3), 5-FU 3 μM; 5-FU(15), 5-FU 15 μM. (C) Colony formation (%) represents the average of three independent experiments, each performed in duplicate, with error bars showing the SE of triplicate experiments. Student’s *t*-test, # *p* = 0.0840, ## *p* = 0.0828, and one-way ANOVA, *p* < 0.0001 (for all groups). (D) 5-FU efficacy (%) indicates the enhancement of 5-FU efficacy in HCT116R^F10^ cells and HCT116 cells, respectively. 5-FU efficacy was calculated using the values for colony formation: 5-FU efficacy (%) = (NSsi + 5-FU – TSsi + 5-FU)/(NSsi alone – TSsi alone) × 100. White bar, *P*: parental HCT116 cells; black bar, R: 5-FU-resistant HCT116R^F10^ cells. Student’s *t*-test, # *p* = 0.2273 (vs *P*) and F-test *p* = 0.0219 (vs *P*).

First, to elucidate the relationship between 5-FU resistance and *TYMS* expression, we analyzed the anticancer activity of 5-FU in the 5-FU-resistant HCT116R^F10^ cells and parental HCT116 cells transfected with *TYMS*-targeted siRNA. HCT116 and HCT116R^F10^ cells were treated with the indicated concentration of 5-FU (EC_20_ values: 3 μM for HCT116 cells; 15 μM for HCT116R^F10^ cells), respectively. Additionally, the knockdown of *TYMS* enhanced the anticancer activity of 5-FU in both types of CRC cells (Figure 2A–C). In the parental HCT116 cells, the percentage of colony formation following 5-FU treatment was lower when the cells were transfected with *TYMS*-targeted siRNA (28%) than with nonsilencing siRNA (55%) (Figures 2A,C). Similarly, in 5-FU-resistant HCT116R^F10^ cells (Figures 2B,C), the percentage of colony formation after 5-FU treatment was lower after transfection with *TYMS*-targeted siRNA (51%) than with nonsilencing siRNA (79%). The enhancement of the anticancer effect of 5-FU cytotoxicity by *TYMS* knockdown was stronger in HCT116R^F10^ cells (186%) than in parental HCT116 cells (50%) (Figure 2D). There are numerous reports that the phenotype of 5-FU sensitivity and resistance is influenced by the levels of TS protein and enzymatic activity in cancer cells.^13−15,18^ These observations suggest that the TS protein’s intracellular abundance, status, and function are important for the phenotypic characteristics of sensitivity and resistance to 5-FU in cancer cells.

### Trapping of FdUMP by the TS Protein is More Effective in 5-FU-Resistant HCT116R^F10^ Cells than in Parental HCT116 Cells

**Figure 3:**
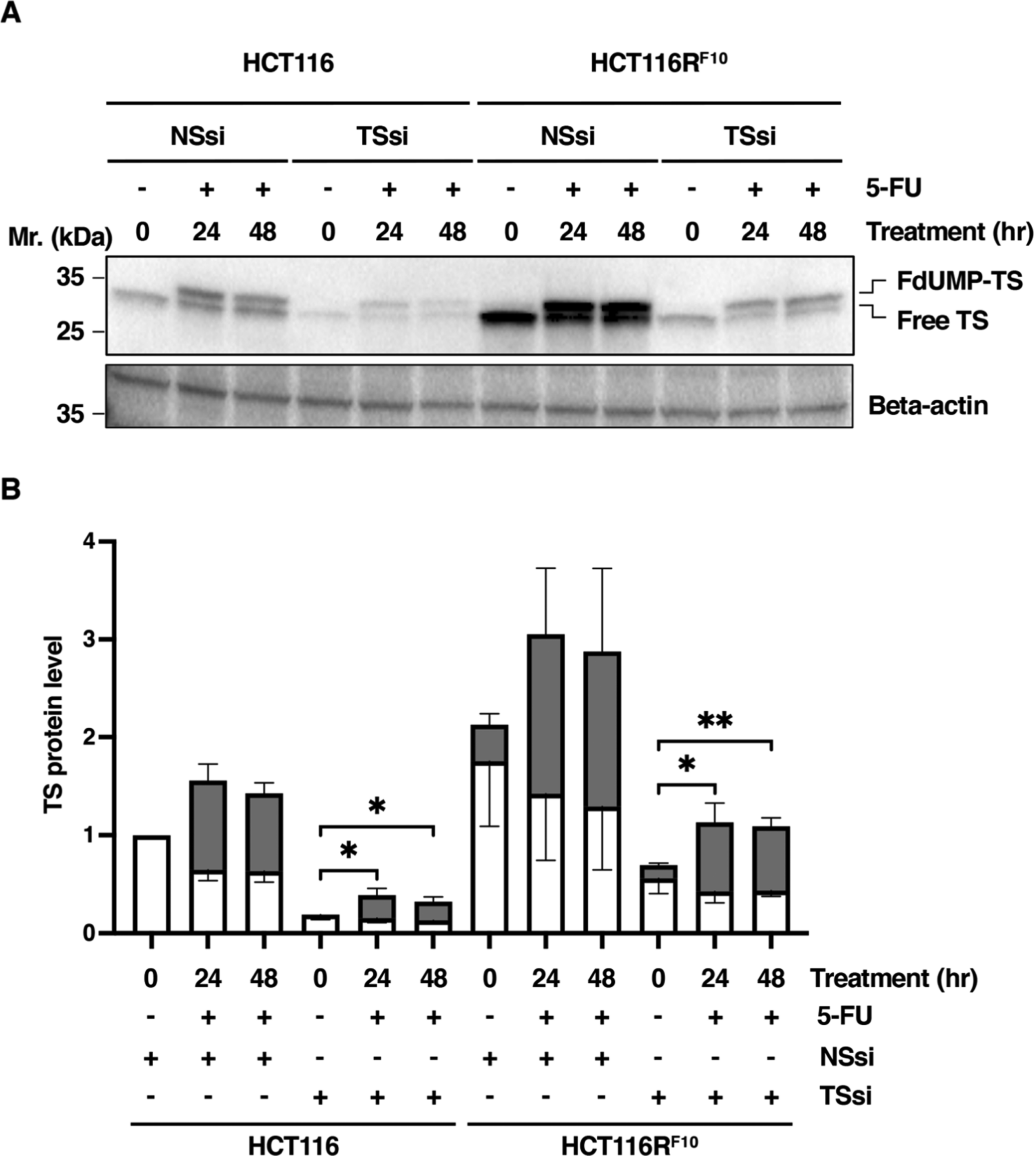
Trapping efficiency of FdUMP by TS is higher in HCT116R^F10^ cells than in parental HCT116 cells. (A) Dynamics of the TS protein in *TYMS*-silenced HCT116R^F10^ cells and HCT116 cells after treatment with 5-FU. At 48 h after transfection with *TYMS*-targeted siRNA or nonsilencing siRNA, the cells were treated with 5-FU 100 μM for the indicated treatment time, and whole-cell lysates were prepared. The protein expression of TS and β-actin was measured by Western blotting analysis. The data are representative of at least three independent experiments. NSsi, nonsilencing siRNA; TSsi, *TYMS*-targeted siRNA; (B) TS protein level in HCT116R^F10^ cells and HCT116 cells. The levels of total TS, i.e., the active free-TS form and the inactive FdUMP-TS form, are indicated by the ratio of TS density to β-actin density for each treatment relative to the value for the NSsi-transfected parental HCT116 cells without 5-FU. The results represent the average of three independent experiments and the error bars show the ±SE of triplicate experiments. White bar, free-TS form; gray bar, FdUMP-TS form. Student’s *t*-test, * *p* < 0.05 and ** *p* < 0.01, one-way ANOVA, *p* < 0.001 (for total TS levels of all groups), and *p* < 0.05 (for FdUMP-TS levels of all groups).

We tested the hypothesis that the TS protein is utilized to trap FdUMP, which results in resistance to 5-FU. As shown in Figure 3A,B, the expression of *TYMS* in untreated and 5-FU-treated parental HCT116 cells and 5-FU-resistant HCT116R^F10^ cells was suppressed by transfection of *TYMS*-targeted siRNA. In the untreated stage, the knockdown efficacies of the TS protein were 86% in HCT116 cells and 63% in HCT116R^F10^ cells transfected with *TYMS*-targeted siRNA compared to that in both cells transfected with nonsilencing siRNA, respectively. The other control experiment, in which nonsilencing siRNA was transfected, showed no effect on the expression of TS and β-actin in either cell type. Similarly, the transfection of *TYMS*-targeted siRNA in both types of cells showed no impact on the expression of β-actin. These control experiments showed similar protein levels of TS and β-actin in HCT116 cells and HCT116R^F10^ cells under both the passage culture condition and 5-FU-treated condition (Figure 1D). In both types of nonsilencing siRNA-transfected TS, i.e., total TS, appears to be overproduced in HCT116R^F10^ cells compared with the parental HCT116 cells with and without 5-FU treatment. The same results were observed when both cell types were transfected with *TYMS*-targeted siRNA. The induction of TS after treatment with 5-FU for 24 h was higher in parental HCT116 cells (1.7-fold increase in NSsi-transfected cells and 2.1-fold increase in TSsi-transfected cells) than in the 5-FU-resistant HCT116R^F10^ cells (1.4-fold increase in NSsi-transfected cells and 1.5-fold increase in TSsi-transfected cells). Furthermore, the accumulation of the FdUMP-TS protein after 5-FU for 24 h was dramatically increased in HCT116R^F10^ cells (1.8–3.0-fold higher) compared with HCT116 cells transfected with nonsilencing siRNA or *TYMS*-targeted siRNA. It is known that the FdUMP-TS protein band, indicating the FdUMP-covalent form, represents TS in ternary complexes and is correlated with the intracellular concentration of FdUMP.^19−22^ Similarly, the storage of active free-TS protein after 5-FU for 24 h was significantly increased in HCT116R^F10^ cells (2.5–2.9-fold higher) compared with HCT116 cells after transfection of nonsilencing siRNA or *TYMS*-targeted siRNA. Notably, the expression of free-TS protein in 5-FU-resistant HCT11R^F10^ cells was decreased (19% at 24 h and 26% at 48 h in NSsi-transfected cells; 23% at 24 h and 23% at 48 h in TSsi-transfected cells) by 5-FU treatment compared with no treatment after transfection of *TYMS*-targeted siRNA or nonsilencing siRNA, respectively. Similarly, the expression of free-TS protein in parental HCT116 cells was decreased (36% at 24 h and 37% at 48 h in NSsi-transfected cells; 21% at 24 h and 32% at 48 h in TSsi-transfected cells) by 5-FU treatment compared with the untreated control after transfection *TYMS*-targeted siRNA or nonsilencing siRNA. These observations indicate that the regulation of the balance between the storage of active free-TS and the accumulation of FdUMP-TS is a leading cause of direct resistance to 5-FU.

## Discussion

TS, which is encoded by the *TYMS* gene in humans, catalyzes the conversion of dUMP to dTMP using the co-substrate CH2-THF as a methyl donor.^5^ The TS enzyme is believed to exist in two forms, a monomer and a dimer, which are in monomer–homodimer equilibrium.^5^ The TS dimer is essential for its catalytic activity. It is known that binding of TS, in its dimeric form, to its own mRNA leads to the formation of an autoregulatory feedback loop that represses the translation of *TYMS* mRNA.^19,23−26^ Many mechanisms have been proposed to explain 5-FU resistance in cancer cells. One important mechanism is the disruption of the autoregulatory feedback loop for the repression of translation. TS ligands, such as 5-FU, disrupt the binding of the TS enzyme with *TYMS* mRNA, leading to translational derepression and overproduction of the TS enzyme.^19,25,26^ In addition to translational derepression, enzyme stabilization has been suggested as the primary mechanism of TS induction by fluoropyrimidines in human CRC and ovarian cancer cell lines.^27−29^ Furthermore, it is proposed that fluoropyrimidine-mediated increases in TS levels are induced by its effect on TS enzyme stability, with no effect on *TYMS* mRNA.^28,30,31^ The amplification of *TYMS*, leading to the overproduction of *TYMS* mRNA and TS protein, is another mechanism of resistance to fluoropyrimidines like 5-FU and its derivatives.^12^ These observations indicated that an understanding of translational derepression, enzyme stabilization, and gene amplification as the process of TS induction can help to elucidate the mechanism of the acquisition of 5-FU resistance. These findings clearly suggest that the mechanisms of 5-FU resistance are a complex and serious problem.

Recently, we established a 5-FU-resistant cell line, HCT116R^F10^ cells, from parental human CRC HCT116 cells and analyzed the resistance mechanisms of 5-FU.^16^ In previous findings, HCT116R^F10^ cells were weakly sensitive to SN-38, the active metabolite of irinotecan, and cisplatin compared with the parental HCT116 cells.^16^ The sensitivity of SN-38 and cisplatin was 1.4-fold (EC_50_ = 3 nM in HCT116R^F10^ cells; 4.2 nM in HCT116 cells) and 1.2-fold (EC_50_ = 4.5 μM in HCT116R^F10^ cells; 5.2 μM in HCT116 cells) higher in HCT116R^F10^ cells than in parental HCT116 cells, respectively.^16^ Additionally, the parental HCT116 cells grow with a doubling time of approximately 18 h. In contrast, 5-FU-resistant HCT116R^F10^ cells grow with a doubling time of approximately 27 h in both passage culture conditions with and without 10 μM 5-FU. Interestingly, the 5-FU-resistant HCT116R^F10^ cells exhibited a lower ability to form colonies and tumor spheres compared with parental HCT116 cells in colony formation and three-dimensional cell culture experiments.^16^ We consider that the difference of proliferation capacity and clonogenicity may be less relevant to anticancer drug sensitivity in HCT116R^F10^ cells and HCT116 cells. Further, we previously reported that 5-FU-resistant HCT116R^F10^ cells have increased *TYMS* expression relative to 5-FU-sensitive parental HCT116 cells and they use a fraction of TS to trap FdUMP, thereby resulting in resistance to 5-FU and its derivative fluorodeoxyuridine.^16^

In this study, we demonstrated that the regulation of the balance between the storage of active free-TS and the accumulation of inactive FdUMP-TS is responsible for the resistance to 5-FU. Our findings suggest that the TS enzyme in 5-FU-resistant HCT116R^F10^ cells can actively and efficiently trap FdUMP. Notably, several studies have shown that 5-FU treatment enhances TS enzyme induction, mainly the ternary complex among TS, FdUMP, and CH2-THF in various human CRC cells and tissues.^1,2,12,32−34^ Indeed, the expression levels of *TYMS* mRNA and TS protein are molecular biomarkers predicting tumor sensitivity to 5-FU.^1,2^ Additionally, 5-FU resistance is associated with the level of TS protein and enzymatic activity in several human CRC cells and tumors.^1,2,12,32^ The numerous findings may support the hypothesis that the trapping of FdUMP by TS enzyme confers resistance to 5-FU and its derivatives, in that several CRC cells and patients with high TS levels are less sensitive to 5-FU. However, it is critical that many studies to date have not discussed the relationship between the FdUMP trapping capacity by TS enzyme, i.e., FdUMP-TS level at total TS level, and the anticancer sensitivity to 5-FU in human CRC cells. Previously, many researchers understood that 5-FU exerts its anticancer effects through inhibition of TS by its active metabolite FdUMP and incorporation of 5-FU’s metabolites, i.e., FUMP and FdUMP, into RNA and DNA, respectively. In particular, we realize that the main anticancer mechanism of 5-FU is inhibiting TS by its active metabolite, FdUMP. In this study, particularly, our findings suggest that the TS enzyme, which is the target of FdUMP, acts as a resistance factor that traps FdUMP in 5-FU-resistant HCT116R^F10^ cells. We think that additional studies in several 5-FU-resistant human CRC cells are needed to understand the mechanisms of 5-FU resistance utilizing the trap of FdUMP by the TS enzyme. We also consider that the regulatory mechanisms of monomeric and dimeric TS protein form differ between 5-FU-resistant HCT116R^F10^ cells and the 5-FU-sensitive parental HCT116 cells. We further investigated the relationship between the regulation of TS protein status, i.e., the balance between active form of free-TS and the inactive TS form (FdUMP-TS–CH2-THF), and that the potential regulators of 5-FU resistance include TS-interacting proteins, mRNAs, and noncoding RNAs.

## Conclusions

**Figure 4:**
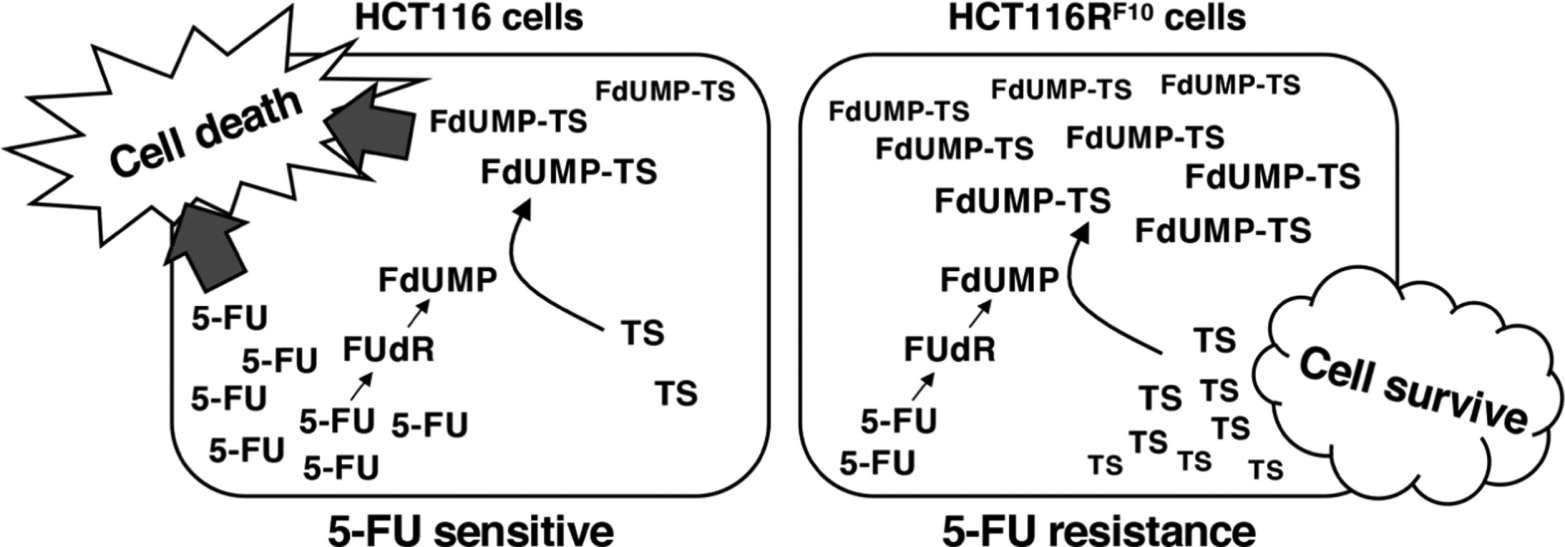
Predictive model of the regulation of TS status by balancing the accumulation of the inactive FdUMP-TS form and the storage of the active free-TS form in the 5-FU-resistant HCT116R^F10^ cells and parental HCT116 cells. We show that the trapping of FdUMP by TS enzyme is more effective in 5-FU-resistant HCT116R^F10^ cells than in parental HCT116 cells. In addition, we predict that the regulation of the balance between the storage of the active TS form and the accumulation of FdUMP-TS is responsible for direct resistance to 5-FU. 5-FU, 5-fluorouracil; FUdR, 5-fluorodeoxyuridine; FdUMP, 5-fluorodeoxyuridine monophosphate; TS, thymidylate synthase.

Collectively, we demonstrated that the trapping of FdUMP by its target enzyme TS confers resistance to 5-FU. In addition, we showed that 5-FU-resistant HCT116R^F10^ cells became resistant to 5-FU by regulating the balance between the storage of the active TS protein and the accumulation of FdUMP-TS protein. In contrast, parental HCT116 cells are sensitized to 5-FU by the depletion of TS, which is due to the formation of the FdUMP-TS complex (Figure 4). Our findings provide a better understanding of the mechanisms of 5-FU resistance and may lead to the development of anticancer strategies to reverse sensitivity to 5-FU and its derivatives.

## Materials and Methods

### Reagents

5-FU was purchased from FUJIFILM Wako Pure Chemical (Osaka, Japan) and stored as a 100 mM stock in dimethyl sulfoxide (DMSO; Sigma-Aldrich, Merck KGaA, Darmstadt, Germany) at −25 °C. The *TYMS*-targeted siRNA (Hs_TYMS_3 FlexiTube siRNA, catalog number: SI00021616, sequence: unpublished) and nonsilencing siRNA (AllStars negative control siRNA, catalog number: 1027280, sequence: unpublished) were obtained from QIAGEN (Dusseldorf, Germany) and stored as a 20 μM stock solution in RNase-free water at −25 °C. Invitrogen Lipofectamine RNAiMax reagent was purchased from Thermo Fisher Scientific (Waltham, MA).

### Cell Lines and Cell Culture

The human CRC cell line HCT116 was obtained from the American Type Culture Collection (Manassas, VA). 5-FU-resistant HCT116 (HCT116R^F10^) cells were produced in accordance with a previously described method.^16^ The parental HCT116 and 5-FU-resistant HCT116R^F10^ cell lines were then cultured as previously described.^16^ Both the parental HCT116 cells and the 5-FU-resistant HCT116R^F10^ cells were grown in Dulbecco’s modified Eagle’s medium (D-MEM, Cat#:043-30085, FUJIFILM Wako Pure Chemical). The culture medium contained 10% heat-inactivated fetal bovine serum, 100 units/mL penicillin, and 100 μg/mL streptomycin.

### Transfection

The transfection of *TYMS*-targeted siRNA (TSsi) or nonsilencing siRNA (NSsi) was performed using the Lipofectamine RNAiMax reagent (Thermo Fisher Scientific) in accordance with the manufacturer’s protocol. Briefly, cells were seeded into six-well plates (5 × 10^4^ cells/well) and then incubated overnight. Prior to transfection, the culture medium was exchanged for 1 mL/well Opti-MEM (Thermo Fisher Scientific). The cells were transfected with TSsi or NSsi (each at 10 nM final concentration). At 4–6 h after transfection, the medium was removed and replaced with an antibiotic-free culture medium.

### Colony Formation Assay

The colony formation assay was performed in accordance with a previously described method.^16,35,36^ The cells were detached using Accutase, suspended in medium, inoculated into six-well plates (200 cells/well), and incubated overnight. Experiments were performed in triplicate. The cells were treated with various concentrations of 5-FU or with solvent (i.e., DMSO) as the negative control. After incubation for 10 days, the cells were fixed with 4% formaldehyde solution, stained with 0.1% (w/v) crystal violet, and the number of colonies in each well was counted. In the transfection experiments, the cells were transfected with TSsi or NSsi (10 nM, as above). After incubation for 24 h, the cells were treated with various concentrations of 5-FU or with DMSO. After incubation for 9 days, the colonies were fixed, stained, and counted.

### Cell Activity by WST-8 Assay

Cell activity assays were performed as previously described.^16^ Cell activity was determined using the Cell Counting Kit-8 (WST-8) cell proliferation assay (Dojindo, Tokyo, Japan).

### Western Blotting

Western blotting analysis was performed as previously described.^16,35^ The antibodies used were rabbit anti-thymidylate synthase (D5B3) monoclonal antibody (9045S, 1:1000, Cell Signaling Technologies, Massachusetts), mouse anti-DPYD (A-5) monoclonal antibody (sc-376712, 1:1000, Santa Cruz Biotechnology, Texas), mouse anti-β-actin monoclonal antibody (A19178-200UL, 1:20 000, Sigma-Aldrich), horseradish peroxidase-linked antirabbit IgG (1:20 000, GE Healthcare, Connecticut), and horseradish peroxidase-linked whole-antibody antimouse IgG (1:20 000, GE Healthcare).

### Statistical Analysis

Statistical analyses were performed using GraphPad Prism 9 software. The data are presented as the mean ± standard error. Significant differences among groups were evaluated using Student’s *t*-test, *F*-test, and one-way analysis of variance (ANOVA). A *p* value of <0.05 was considered to indicate statistical significance.
